# Effect of phospholipid head group on ultrasound-triggered drug release and cellular uptake of immunoliposomes

**DOI:** 10.1038/s41598-023-43813-4

**Published:** 2023-10-03

**Authors:** Nahid S. Awad, Vinod Paul, Nour M. AlSawaftah, Ghaleb A. Husseini

**Affiliations:** 1 Department of Chemical and Biological Engineering, American University of Sharjah, Sharjah, United Arab Emirates; 2 Materials Science and Engineering Program, American University of Sharjah, Sharjah, United Arab Emirates

**Keywords:** Biophysics, Cancer, Drug discovery

## Abstract

Liposomes are the most successful nanoparticles used to date to load and deliver chemotherapeutic agents to cancer cells. They are nano-sized vesicles made up of phospholipids, and targeting moieties can be added to their surfaces for the active targeting of specific tumors. Furthermore, Ultrasound can be used to trigger the release of the loaded drugs by disturbing their phospholipid bilayer structure. In this study, we have prepared pegylated liposomes using four types of phospholipids with similar saturated hydrocarbon tails including a phospholipid with no head group attached to the phosphate head (DPPA) and three other phospholipids with different head groups attached to their phosphate heads (DPPC, DPPE and DPPG). The prepared liposomes were conjugated to the monoclonal antibody trastuzumab (TRA) to target the human epidermal growth factor receptor 2 (HER2) overexpressed on HER2-positive cancer cells (HER2+). We have compared the response of the different formulations of liposomes when triggered with low-frequency ultrasound (LFUS) and their cellular uptake by the cancer cells. The results showed that the different formulations had similar size, polydispersity, and stability. TRA-conjugated DPPC liposomes showed the highest sensitivity to LFUS. On the other hand, incubating the cancer cells with TRA-conjugated DPPA liposomes triggered with LFUS showed the highest uptake of the loaded calcein by the HER2+ cells.

## Introduction

Chemotherapeutic drugs are commonly used to treat different types of cancer. They circulate in the body to destroy cancer cells by preventing them from growing and dividing. Collectively, these drugs are highly toxic with low specificity, targeting both healthy and diseased fast-growing cells indiscriminately. Therefore, they are associated with multiple side effects that significantly lower the patient’s quality of life. Certain side effects are serious such as kidney failure and severe cardiotoxicity^1,2^. These side effects limit the dose that can be administered, reducing drug efficiency, primarily because most anti-neoplastic agents are rapidly metabolized and eliminated from the body^3,4^. One of the promising methods to address this limitation is to encapsulate chemotherapeutic agents inside nano-sized carriers which transport these drugs while reducing their contact with healthy cells. Liposomes present the most successful nanocarrier developed to date with several liposomal formulations already approved and available commercially to treat different types of cancer, such as Doxil^®^, Onivyde™, Depocyt^®^, Visudyne^®^ and Marqibo^®^^5^.

**Figure 1:**
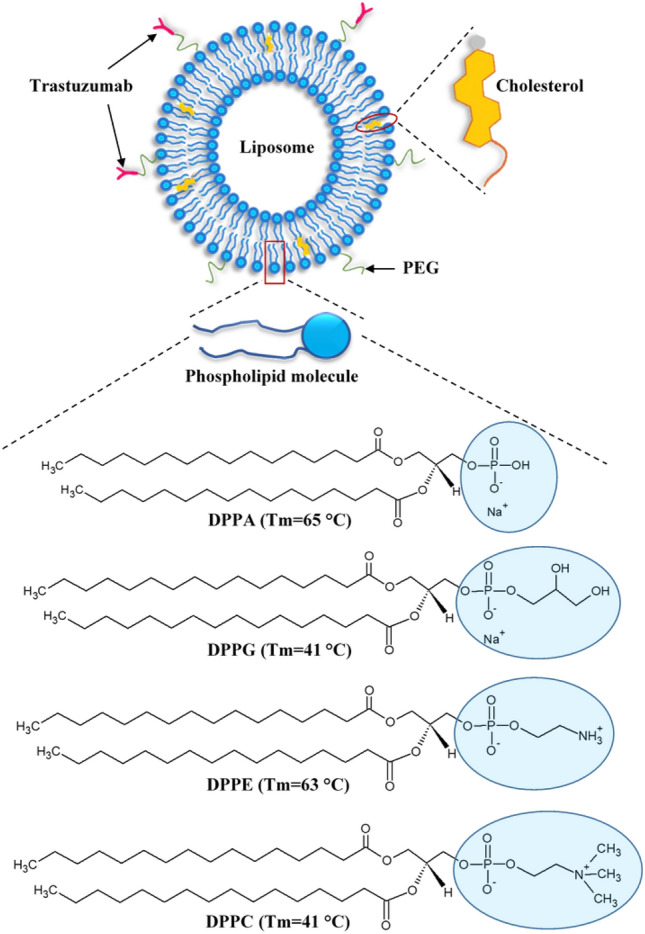
The different phospholipids used to prepare the phospholipid bilayer of the liposomes.

Liposomes are artificial nano-sized vesicles composed of amphipathic membrane made up of phospholipid molecules. Phospholipids occur naturally, forming the bilayer cellular membranes of all living organisms. Thus, the use of phospholipids in liposomal formation makes them physiologically acceptable and highly biocompatible. Phospholipids are bipolar molecules consisting of a hydrophilic (polar) head containing a phosphate group bound to two hydrophobic (non-polar) tails of fatty acid chains esterified with glycerol. When placed in an aqueous solution, phospholipid molecules form bilayer vesicles where the polar heads, which contain a negatively charged phosphate group covalently bonded to a small organic molecule, are aligned towards the outer and inner aqueous phase, while the hydrophobic tails are positioned within the lipid bilayer^6^. The formed bilayer vesicles have a large aqueous interior that can incorporate hydrophilic drugs and a lipid bilayer membrane suitable for loading hydrophobic drugs. Liposomes can be prepared using natural or synthetic phospholipids. Phospholipids commonly used in liposomes formation are phosphatidylcholines (PC) with choline as a head group, phosphatidylglycerols (PG) with glycerol as a head group, phosphatidylethanolamines (PE) with ethanolamine as a head group and phosphatidic acids (PA) with no head group attached to the phosphate head. PC and PE are zwitterionic, i.e., they carry both a positive and a negative charge within the same head group and, thus, have an overall neutral charge at the physiological pH, whereas PG and PA are negatively charged at the physiological pH. Figure 1 depicts the liposomal structure and phospholipids used to prepare them together with their transition temperature (T_m_). T_m_ is the temperature at which the fatty acid chains change from a closely packed and ordered gel phase to a random and disordered liquid phase^7,8^. Liposomes are commonly prepared using phospholipids characterized by high transition temperatures. This choice is primarily due to their enhanced stability at physiological temperatures, improving drug retention and enhancing control over drug release. Phospholipids with high transition temperatures are less likely to prematurely release their encapsulated contents, making them more suitable for the conjugation of targeting moieties to liposomes due to their heightened stability^9^.

Incorporating inert biocompatible polymeric chains, such as polyethylene glycol (PEG), into the liposomes' surface enhances their circulation time in the blood by impairing their recognition by opsonin proteins^10^. The small size of the liposomes allows them to benefit from the leaky nature of the blood vessels surrounding tumors^11^ and the dysfunctional lymphatic system; thus, liposomes can extravasate through the blood vessels and accumulate inside tumoral tissues, a phenomenon known as the enhanced permeability and retention (EPR) effect^12^. The accumulation of liposomes at tumor sites due to the EPR effect is known as passive targeting, which strongly depends on the small size of the liposomes. To enhance the selectivity of the liposomes to the targeted cancer cells, active targeting of tumors can be achieved by decorating the surface of the liposomes with different types of targeting ligands such as small molecules, proteins, peptides, carbohydrates, and antibodies. These targeting ligands can bind to the overexpressed receptors located on the cancer cells' surface, thus minimizing the off-target effects^13–15^.

Once they reach their target site, the encapsulated drug must be released in a controlled manner from the liposome. Several internal and external triggering mechanisms can be exploited to stimulate the release of drugs encapsulated in liposomes; these can be broadly categorized into internal and external triggering mechanisms^16,17^. Liposomes that are sensitive to internal stimuli are designed to benefit from the unique characteristics of the tumor microenvironment, i.e., elevated temperature, lower pH, and a reducing environment; these include thermosensitive liposomes^18^, pH-sensitive liposomes^19^ and enzyme-responsive liposomes^20^. External triggers can also be employed to enhance drug release from the loaded liposomes, such as light^21^, magnetic, and electric fields^22,23^. In addition to those external triggers, the energy generated from ultrasound waves can be employed as an effective triggering mechanism to secure a fast and controllable drug release within tumors^24^.

Ultrasound is used in the medical field as a safe and non-invasive imaging and treatment tool that can deeply penetrate different tissues. Ultrasound can also be focused to target specific sites inside the body. The energy produced from ultrasonic waves, as they propagate through a medium, is absorbed, causing an increase in temperature; this is the thermal effect of ultrasound. Furthermore, as sound waves travel through a medium, they create areas of compression (high pressure) and rarefaction (low pressure), which result in the formation of gas-filled bubbles (cavitation nuclei) that oscillate and could eventually collapse. This is the mechanical effect of ultrasound and is known as acoustic cavitation. Cavitation can be either stable cavitation, where the formed bubbles oscillate about a certain equilibrium size (small amplitude), or inertial cavitation, where the bubbles grow to a large size (large amplitude) and eventually collapse violently^25,26^. Both mechanical and thermal effects play a role in the ability of ultrasound to trigger drug release from liposomes.

Cancer cells expressing HER2 protein (HER2+) are known for faster growth rates compared to HER2-negative cells. In a previous study conducted by our group, we compared drug release, as well as drug uptake by the cancer cells from pegylated DPPC liposomes with and without conjugation to Trastuzumab (TRA), a humanized monoclonal antibody specifically targeting HER2 receptors. Additionally, we explored the effect of LFUS on drug release and drug uptake by the cells from both the non-conjugated (control) and TRA-conjugated DPPC liposomes. Our findings indicated that TRA conjugation led to a significant enhancement in the liposomes’ sensitivity to sonication and drug uptake by the HER2+ cancer cells, which was further enhanced when low-frequency ultrasound (LFUS) was applied^27^. In the present study, we shift our research focus to explore alternative phospholipids that bear similarities to DPPC phospholipids in their hydrocarbon tails but differ solely in their head groups. The aim is to determine if alternative liposomal formulations might yield more favorable outcomes than DPPC, in terms of drug release and cellular uptake, when studied in their pure liposomal form (without TRA conjugation) and following TRA conjugation while also considering the influence of LFUS in both scenarios.

## Materials and methods

### Materials

1,2-Dipalmitoyl-sn-glycero-3-phosphate (DPPA), 1,2-dipalmitoyl-sn-glycero-3-phosphocholine (DPPC), 1,2-dipalmitoyl-sn-glycero-3-phosphoethanolamine (DPPE), 1,2-dipalmitoyl-sn-glycero-3-phospho-(1'-rac-glycerol) (DPPG) and 1,2-distearoyl-sn-glycero-3-phosphoethanolamine-N [amino (polyethylene glycol)-2000] (DSPE-PEG (2000) NH_2_) were obtained from Avanti Polar Lipids Inc. (Alabaster, AL, USA, supplied by Labco LLC. Dubai, UAE). Epithelial breast cancer (HCC1954) cell line was purchased from American Type Culture Collection (ATCC, Manassas, VA, USA), Trastuzumab (Herceptin) was obtained from Hoffmann-La Roche Limited (Basel, Switzerland, supplied by Aster pharmacy, Sharjah, UAE). All the other chemicals used here were obtained from Sigma Aldrich Chemie GmbH (Munich, Germany, supplied by Labco LLC. Dubai, UAE).

### Liposomes preparation

Different phospholipids were used to prepare liposomes using the thin-film hydration method. DPPA, DPPC, DPPE, or DPPG were added to cholesterol and DSPE-PEG(2000)-NH_2_ at molar ratios of 65:30:5, respectively and were dissolved in chloroform/methanol (1:2) in a round bottom flask. The solvent was then evaporated using a rotary evaporator under a vacuum (50 °C) for 15 min to form a thin layer of lipids on the walls of the round bottom flask. The film was then hydrated using 2 ml of a 30-mM calcein solution (pH 7.7) for 50 min at 60 °C (for DPPC and DPPG liposomes) and 70 °C (for DPPA and DPPE liposomes) to form multilamellar vesicles (MLV). Unilamellar vesicles (ULV) were formed by sonication for 2 min using a 35-kHz sonicator bath (Elma D-78224, Melrose Park, IL, USA) at 60 °C for DPPC/DPPG liposomes and 70 °C for DPPA and DPPE liposomes. The resulting liposomes were extruded through polycarbonate filters (pore diameter of 200 nm) mounted in an Avanti Extruder (Avanti Polar Lipids, Inc., Alabaster, AL, USA) at 60 °C for DPPC/DPPG liposomes and 70 °C for DPPA and DPPE liposomes. Unencapsulated calcein was removed using centrifugation (3000 rpm for 1 h), and the pellets were resuspended in 1 ml borate buffer (pH 8.5). TRA conjugation to the liposomes was performed using cyanuric chloride through the temperature base double substitution reaction by adding 9.2 µl of cyanuric chloride (10 mg/ml) to 0.5 ml of deionized water. The solution was then added to 1 ml of the liposomes and was left to stir for 3 h. Next, 1 mg of TRA was dissolved in 0.5 ml borate buffer (pH 8.5) and was added to the liposomes. The mixture was then left to stir overnight. Unconjugated TRA was removed using centrifugation (3000 rpm for 1 h), and the pellets were resuspended in 1 ml PBS buffer (pH 7.4). The prepared liposomes were kept at 4 °C until used. The protein (TRA) was introduced into both the conjugated liposomes (TRA-liposomes) and the non-conjugated liposomes (control liposomes) to ensure a balanced experimental setup. However, the linking agent (cyanuric chloride), which connects TRA molecules to the liposomes, was exclusively included in the TRA-liposomes. In the case of the control liposomes, it was substituted with deionized water.

### Liposomes characterization

The hydrodynamic radius and polydispersity of the prepared liposomes were determined using dynamic light scattering (DLS) (DynaPro NanoStar, Wyatt Technology Corp., Santa Barbara, CA, USA). Samples were diluted (15 μl in 1 ml of PBS), and the aliquot of the mixture was transferred to a cuvette and then inserted in the designated compartment for measurements. Zeta potential measurements of the prepared liposomes were determined using Litesizer 500 (Anton Paar GmbH, Graz, Austria). The phospholipids concentration was measured using the Stewart assay and the protein quantification was performed using the bicinchoninic acid assay (BCA), as described in our previous work^27^. The Stewart assay is based on the formation of a red-colored complex between the phospholipid head groups and ammonium ferrothiocyanate, which is quantified spectrophotometrically (at 485 nm) following its extraction into chloroform. Specific calibration curves are used for the different phospholipid head groups. The BCA assay combines the reduction of Cu^2+^ to Cu^1+^ by protein with the colorimetric detection of the Cu^1+^ cation using a reagent containing bicinchoninic acid. The purple-colored reaction product of this assay is formed by the chelation of two molecules of BCA with one Cu^1+^ cation exhibiting a strong absorbance at 562 nm.

**Figure 2:**
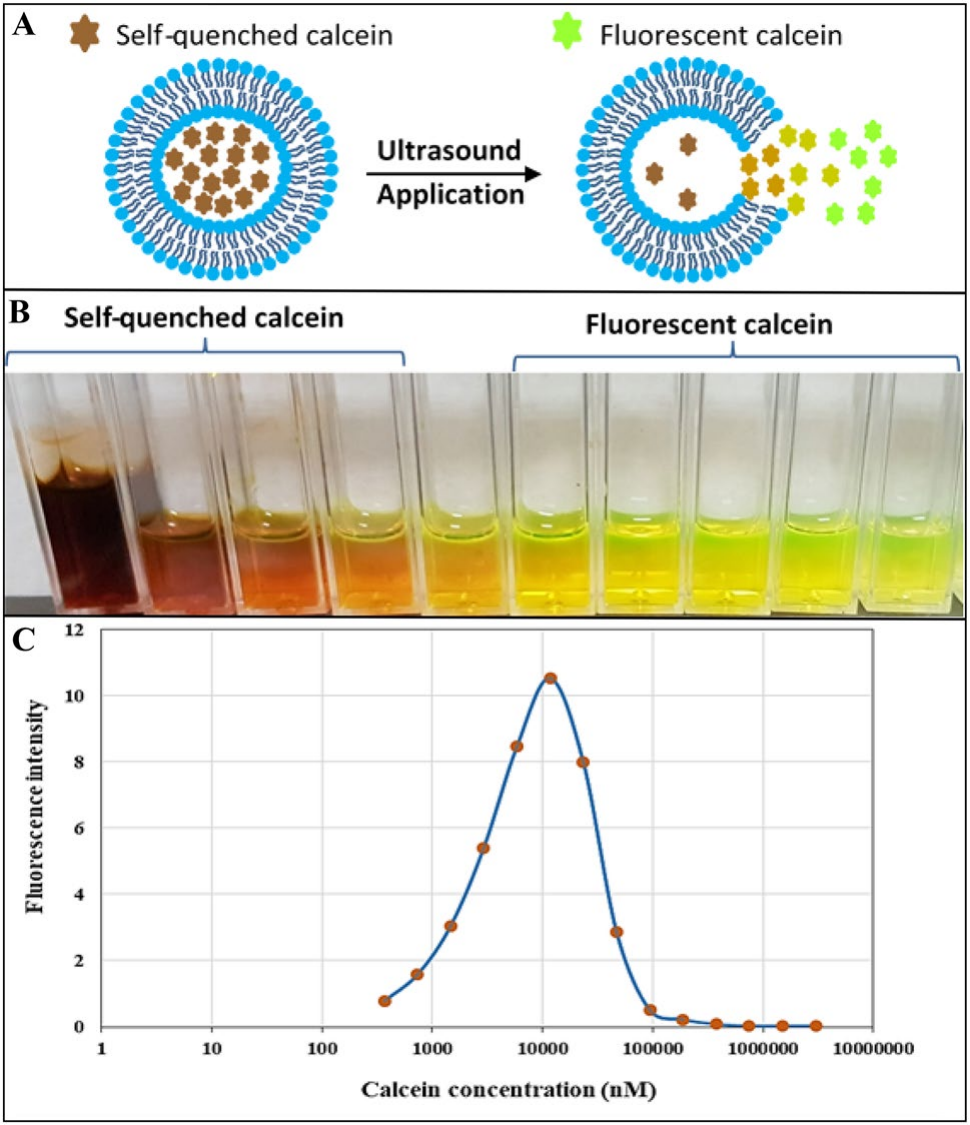
Calcein is self-quenching inside the liposomes and shows fluorescence properties once released and diluted in the surrounding solution (**A**). A serial dilution of calcein from self-quenching concentrations to lower concentrations with fluorescence properties (**B**). Calcein fluorescence intensity as a function of calcein concentration (**C**).

Determination of the encapsulation efficiency of the different formulations was done by monitoring calcein fluorescence readings before and after the addition of Triton X-100, as prescribed in 2.4. The concentration of the encapsulated calcein was determined against a calibration curve prepared using different concentrations of calcein (Fig. 2). The stability of the different formulations of pegylated liposomes was determined by incubating the diluted liposomes (75 µl in 3 ml of PBS) in Fetal Bovine Serum (FBS-10%) for 2 h at 37 °C. Released calcein, before and after the addition of Triton X-100, was monitored using a fluorimeter as described in “Low-frequency ultrasound release studies”. High-resolution transmission electron microscopy (HRTEM) was used to image the synthesized liposomes; the HRTEM used is a Talos F200X Scanning/Transmission Electron Microscope as prescribed in our previous work^28^.

### Low-frequency ultrasound release studies

Samples were prepared by placing 3 ml of PBS buffer in a cuvette, 75 µl of the liposomes were added to the same cuvette and were mixed gently. Calcein release from the liposomes was triggered using a low-frequency ultrasonic (LFUS) probe at 20 kHz (VCX750, Sonics & Materials Inc., Newtown, CT) using a power density of 10 mW/cm^2^ in a pulsed mode (20 s *on* and 10 s *off*). Online monitoring of calcein fluorescence following its release from the liposomes was conducted at room temperature at excitation wavelengths of 495 nm and emission wavelength of 515 nm (slit width of 5 nm) using a spectrofluorometer (Photon Technology International, Edison NJ, USA). The initial fluorescence intensity, *I*_*o*_, was measured for 70 s before sonication. Once a fluorescence plateau was reached, 50 μl of Triton X-100 were added to the sample to lyse the liposomes and achieve 100% calcein release. The following equation was used to calculate the cumulative fraction release (CFR) of calcein:

1 $$CFR=\frac{{I}_{t}-{I}_{o}}{{I}_{\infty }-{I}_{o}}$$

where *I*_0_ stands for calcein fluorescent intensity before applying ultrasound, *I*_*t,*_ stands for calcein fluorescent intensity at time, *t*, and *I*_*∞*_ is the highest recorded calcein fluorescence intensity.

### Flow cytometry analysis

HER2-positive cells (HCC1954 cells) were seeded in 6-well plates (2 × 10^5^ cells/ml) and were incubated in humidified air with 5% CO_2_ at 37 °C for 24 h. Cells were then treated with the different formulations of liposomes and were placed back under the same incubation conditions for 4 h. Next, the plates exposed to ultrasound were placed in a LFUS sonicating bath (35-kHz) for 20 s and were incubated for another hour. The cells were then treated with Trypsin to facilitate the detachment of the cell. Finally, the cells were collected using centrifugation and resuspended in PBS buffer to conduct the flow cytometry analysis.

### Statistical analysis

Results were reported as average ± standard deviation (SD). Liposome sizes, release percentage and flow cytometric analysis were compared using one-way ANOVA tests.

## Results

### Liposomes characterization

Four different phospholipids were used to prepare pegylated liposomes with and without conjugation to TRA molecules as follows: DPPC liposomes (control DPPC) and DPPC liposomes conjugated to TRA (DPPC-TRA), DPPE liposomes (control DPPE) and DPPE liposomes conjugated to TRA (DPPE-TRA), DPPA liposomes (control DPPA) and DPPA liposomes conjugated to TRA (DPPA-TRA) as well as DPPG liposomes (control DPPG) and DPPG liposomes conjugated to TRA (DPPG-TRA). Table 1 shows the prepared liposomes' average size, polydispersity percentage (Pd%) and zeta potentials before and after TRA conjugation. As seen from the results, all liposomal formulations showed no significant difference in size (p = 0.624). Moreover, TRA conjugation resulted in a significant increase in liposomes’ size (p < 0.05). The polydispersity percentage remained within an acceptable range of under 20% for all the tested formulations before and after TRA conjugation.

**Table 1:** Average size (radius) and polydispersity percentage (Pd%) of the different liposomal formulations loaded with calcein.

Phospholipids	Before conjugation			After conjugation		
	Radius (nm)	Pd%	Zeta potential (mV)	Radius (nm)	Pd%	Zeta potential (mV)
DPPE	87.2 ± 0.75	10.3 ± 0.55	− 3.2 ± 0.75	104.1 ± 1.41	15.6 ± 1.87	− 14.9 ± 0.73
DPPC	88.9 ± 1.31	11.7 ± 0.62	− 10.9 ± 0.21	111.7 ± 1.93	13.0 ± 112	− 24.7 ± 1.58
DPPA	89.8 ± 1.64	6.9 ± 0.42	− 20.3 ± 1.21	108.2 ± 0.96	14.7 ± 0.96	− 27.8 ± 0.62
DPPG	85.3 ± 0.50	11.4 ± 1.40	− 26.1 ± 2.34	112.4 ± 1.33	16.1 ± 2.4	− 34.5 ± 1.46

**Figure 3:**
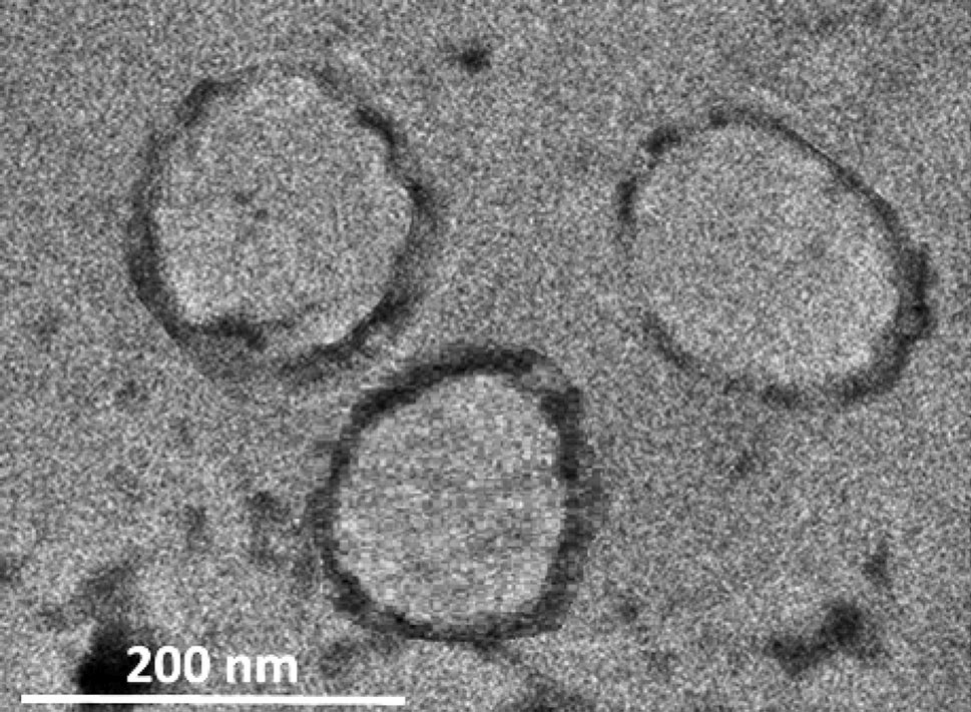
TEM image of DPPC-TRA liposomes.

All the non-conjugated pegylated formulations (controls) showed negative surface charges, with DPPG and DPPE showing the highest and lowest negative charge, respectively (DPPE < DPPC < DPPA < DPPG). TRA conjugation resulted in a significant increase in the negative surface charge of all the tested formulations. The average phospholipid content was (9.3 mg/ml ± 1.21) with no significant difference in phospholipids concentration between the liposomes (p = 0.862). Conjugated liposomes showed a 1.9-fold increase in protein concentration (average = 47 ± 1.27 μg/ml per mg of lipids) compared to that of the control liposomes (24 ± 2.13 μg/ml per mg of lipids). TRA was added to the control liposomes with no conjugation reaction. Figure 3 shows a TEM image of DPPC-TRA liposomes.

On average, all the tested formulations encapsulated 1 ± 0.23 mM of calcein within their cores. From the calibration curve (Fig. 2, calcein fluorescence vs. concentration), at a concentration of 1 mM, calcein is at a self-quenched concentration and will produce no fluorescent properties. Therefore, the loaded liposomes were used as the baseline. When calcein is released into the surrounding solution, the self-quenching is reduced as calcein concentration decreases. The stability of the prepared liposomes was determined by monitoring their ability to retain their load of calcein at the physiological temperature (37 °C) for 2 h. DPPC-TRA, DPPE-TRA, DPPA-TRA and DPPG-TRA liposomes showed similar stability results, releasing on average 3% ± 0.89, 2.4% ± 0.31, 2.6% ± 0.53 and 3.2 ± 0.22% of the encapsulated calcein (p = 0.425) following 2 h of incubation in FBS at 37 °C. All the reported values are the average of three different batches of liposomes.

### Triggering calcein release using low-frequency ultrasound (LFUS)

**Figure 4:**
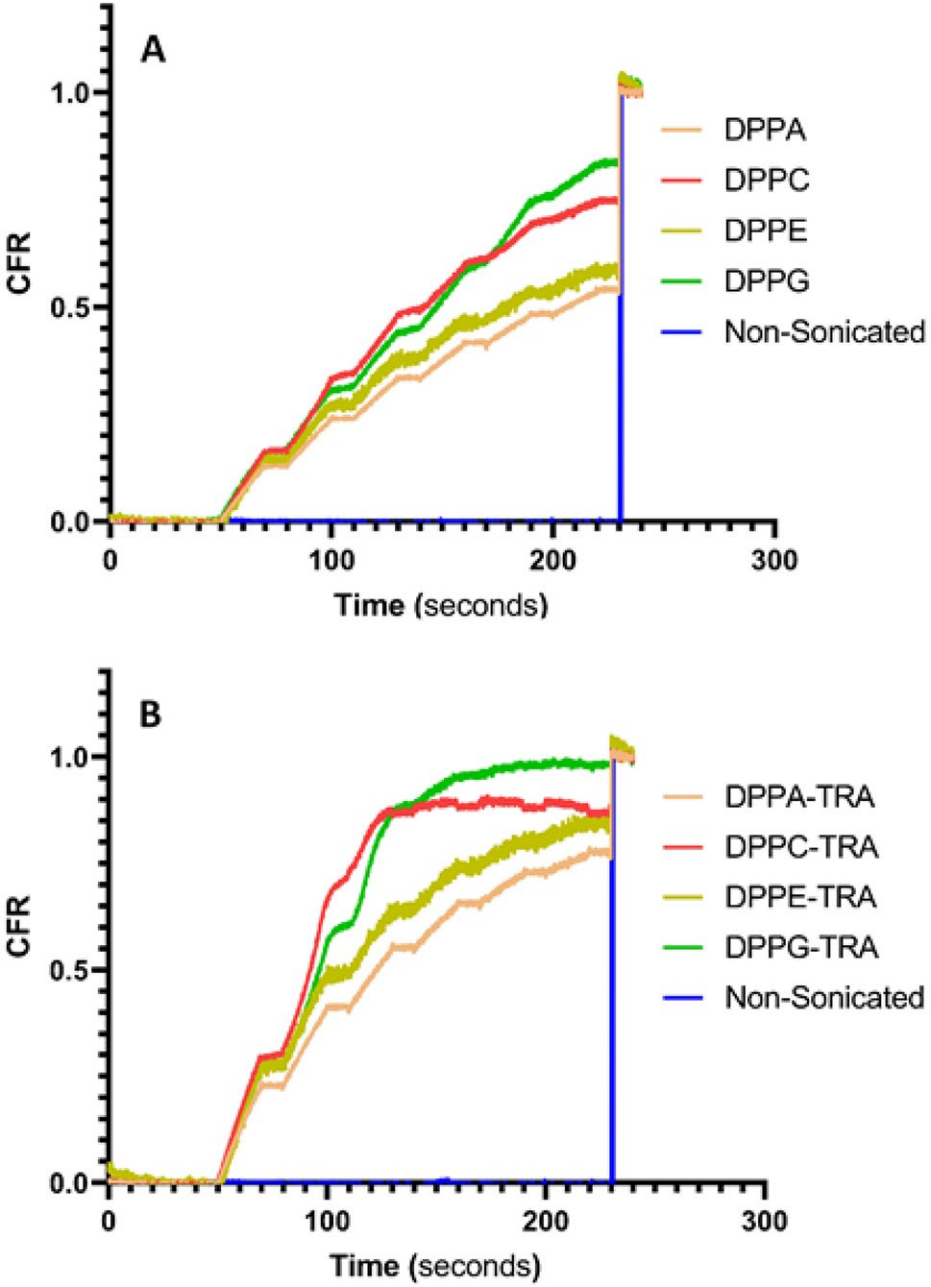
Normalized release profiles of the synthesized non-conjugated (control) liposomes (**A**) and TRA-conjugated liposomes (**B**) loaded with calcein following the exposure to pulsed LFUS at 10 mW/cm^2^. Results are the average of three batches of liposomes with three replicates each.

Calcein release from non-conjugated (control) and TRA-conjugated liposomal formulations was triggered using LFUS (20-kHz US) at 10 mW/cm^2^ in a pulsed mode (20 s *on* and 10 s *off*). The trends of the released calcein were monitored online and the normalized release rates from all the tested liposomes are shown in Fig. 4. All types of liposomes showed a controlled drug release when exposed to pulsed LFUS, exhibiting an increased calcein release during the “*on*” mode of pulsed LFUS and no increase in the release was detected during the “*off*” mode. The pattern continued until a plateau was reached. As seen in Fig. 4A, the non-conjugated (control) formulations showed different responses to LFUS, with DPPC and DPPG liposomes releasing significantly more calcein (54.6% and 52.2%, respectively), following the third pulse of LFUS, compared to DPPA and DPPE liposomes which released 37.7% and 42.9% of the encapsulated calcein, respectively. DPPC and DPPG liposomes exhibited a quicker release rate, reaching a plateau after 130 s and 160 s of pulsed LFUS, respectively. In contrast, the DPPA and DPPE formulations took longer to plateau, requiring 210 s and 220 s of pulsed LFUS, respectively.

**Figure 5:**
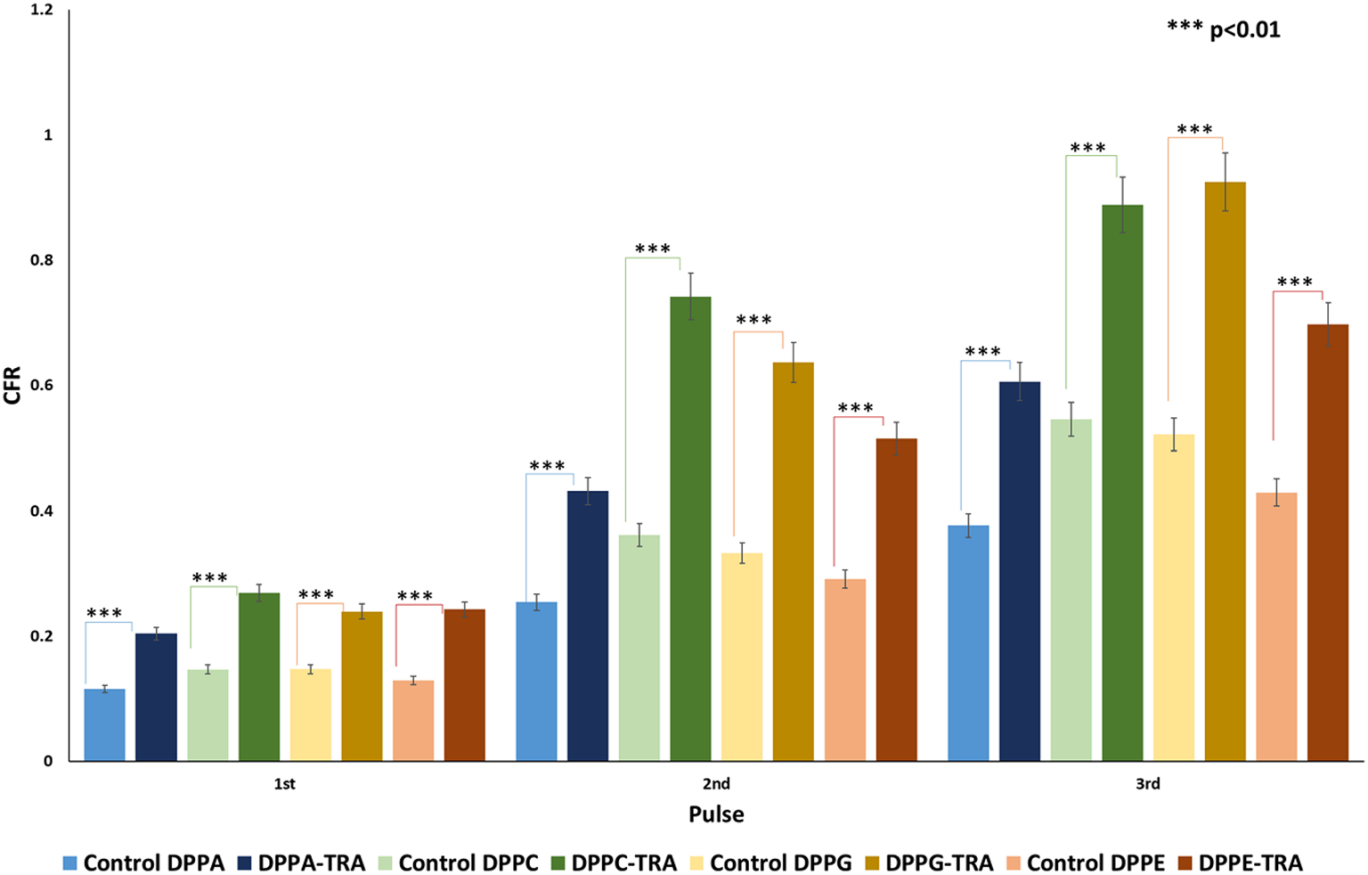
Comparison of calcein release for the first three pulses from non-conjugated and TRA-conjugated liposomes synthesized using different phospholipids at 10 mW/cm^2^ expressed as the CFR defined in Eq. (1).

Interestingly, Fig. 4B showed that TRA conjugation to the liposomes significantly enhanced calcein release from all the tested formulations, with DPPC releasing 88.9% (p = 2.72 × 10^–70^), DPPG releasing 92.5% (p = 1.15 × 10^–86^), DPPA releasing 60.6% (p = 8.28 × 10^–6^), and DPPE releasing 69.8% (p = 2.93 × 10^–61^), following the first three pulses of LFUS. Calcein release from the control liposomes (DPPA, DPPC, DPPE and DPPG) as well as TRA-conjugated liposomes (DPPA-TRA, DPPC-TRA, DPPE-TRA and DPPG-TRA) that were not sonicated with ultrasound was also recorded. As seen in Fig. 4, these liposomes remained intact with no calcein release. The addition of Triton X-100 resulted in lysing the liposomes and releasing all the encapsulated calcein from the non-sonicated liposomal formulations. Figure 5 shows a detailed comparison of calcein release from the different liposomal formulations following the first three pulses.

Finally, the mechanical index (MI), is a parameter used to indicate the possibility of the occurrence of cavitation. The negative pressure (expressed in units of MPa) in the above equation is dependent upon the acoustic impedance of water, *Z,* and the intensity of the LFUS, *I,* (expressed in W/cm^2^). Negative pressure is denoted by Eq. (2).

2 $${P}_{neg}=\sqrt{2 I Z}$$

Since soft body tissues' acoustic impedance is comparable to that of water when determining *P*_*neg*_, the acoustic impedance of water, i.e., 1.48 MPa sec/m was used^29,30^. The LFUS power density used in this work was 10 mW/cm^2^, corresponding to MI values of 0.121. The threshold of collapse cavitation is expected to occur at around MI = 0.3, biological effects are observed at MI > 0.7, and tissue damage is expected to occur at MI > 1^31–34^. Therefore, our experimental MI value is well below the collapse cavitation threshold of 0.3, indicating the occurrence of stable cavitation.

### Flow cytometry analysis of cellular uptake of calcein

**Figure 6:**
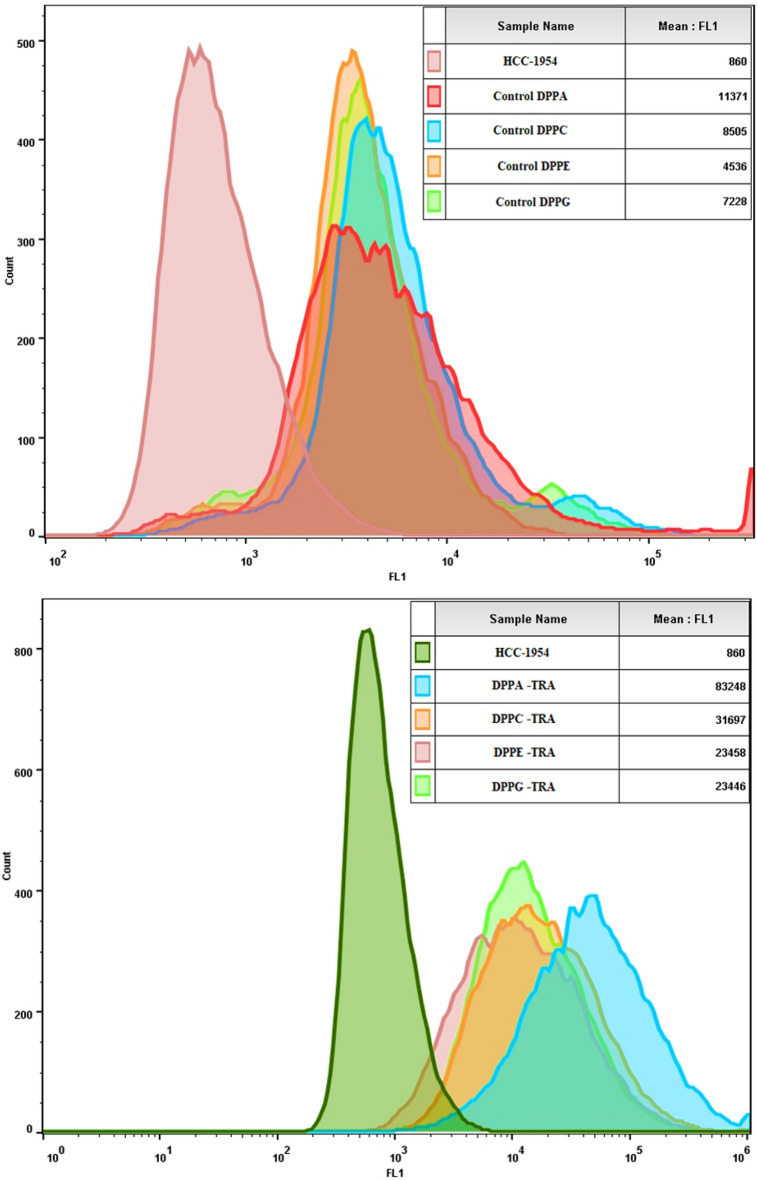
Flow cytometry results showing HCC1954 cellular uptake of calcein after being incubated with the different non-conjugated (controls) formulations of liposomes (Control DPPA. Control DPPC, Control DPPE, Control DPPG) (Top) and TRA-conjugated formulations (DPPC-TRA, DPPE-TRA, DPPA-TRA and DPPG-TRA) (Bottom). Cells with no treatment were used as a negative control for background fluorescence. An average of 2 × 10^5^ cells/ml cells was analyzed from each sample with calcein fluorescence intensity showed on a four-decade log scale.

In our previous study (Elamir et al.^27^), we have established that cellular uptake of calcein encapsulated inside DPPC liposomes conjugated to TRA, by the HER2+ cells was significantly higher when compared to the control liposomes with no TRA conjugation. Furthermore, no significant difference was recorded in calcein uptake by the HER2− cells when incubated with both types of liposomes. In this study, we are comparing calcein uptake by the HER2 positive (HER2+) cell line (HCC1954) from the different formulations of the plain (control) liposomes (DPPA, DPPC, DPPE and DPPG) as well as he conjugated formulations (DPPA-TRA, DPPC-TRA, DPPE-TRA and DPPG-TRA). The cells were incubated with the different formulations of liposomes for 4 h. Following the incubation period, calcein uptake by the cells was then monitored using flow cytometry. Figure 6 shows the fluorescence intensity of calcein inside the cells; cells with no treatment were used as a negative control for background fluorescence. The comparison between non-conjugated (control) liposomes showed that the highest calcein uptake by the cells was recorded following the incubation with control DPPA liposomes (11,371 ± 230 A.U.), which was significantly higher (p = 0.037) compared to the second-highest calcein uptake which was recorded following the incubation with control DPPC liposomes (8505 ± 211 A.U.). Figure 6 also shows that, compared to the non-conjugated counterparts, incubation with TRA-conjugated formulations resulted in a significant increase in calcein uptake by the cells (DPPA-TRA (82,634 ± 1.45 A.U., p = 0.0013), DPPC-TRA (33,188 ± 406 A.U., p = 0.0033), DPPE-TRA (24,631 ± 394 A.U., p = 2.1 × 10^–6^) and DPPG-TRA (24,016 ± 125 A.U., p = 3.2 × 10^–5^). As seen from the results, cells incubated with DPPA-TRA showed a significant increase in calcein uptake compared to the other targeted formulations (p < 0.05).

**Figure 7:**
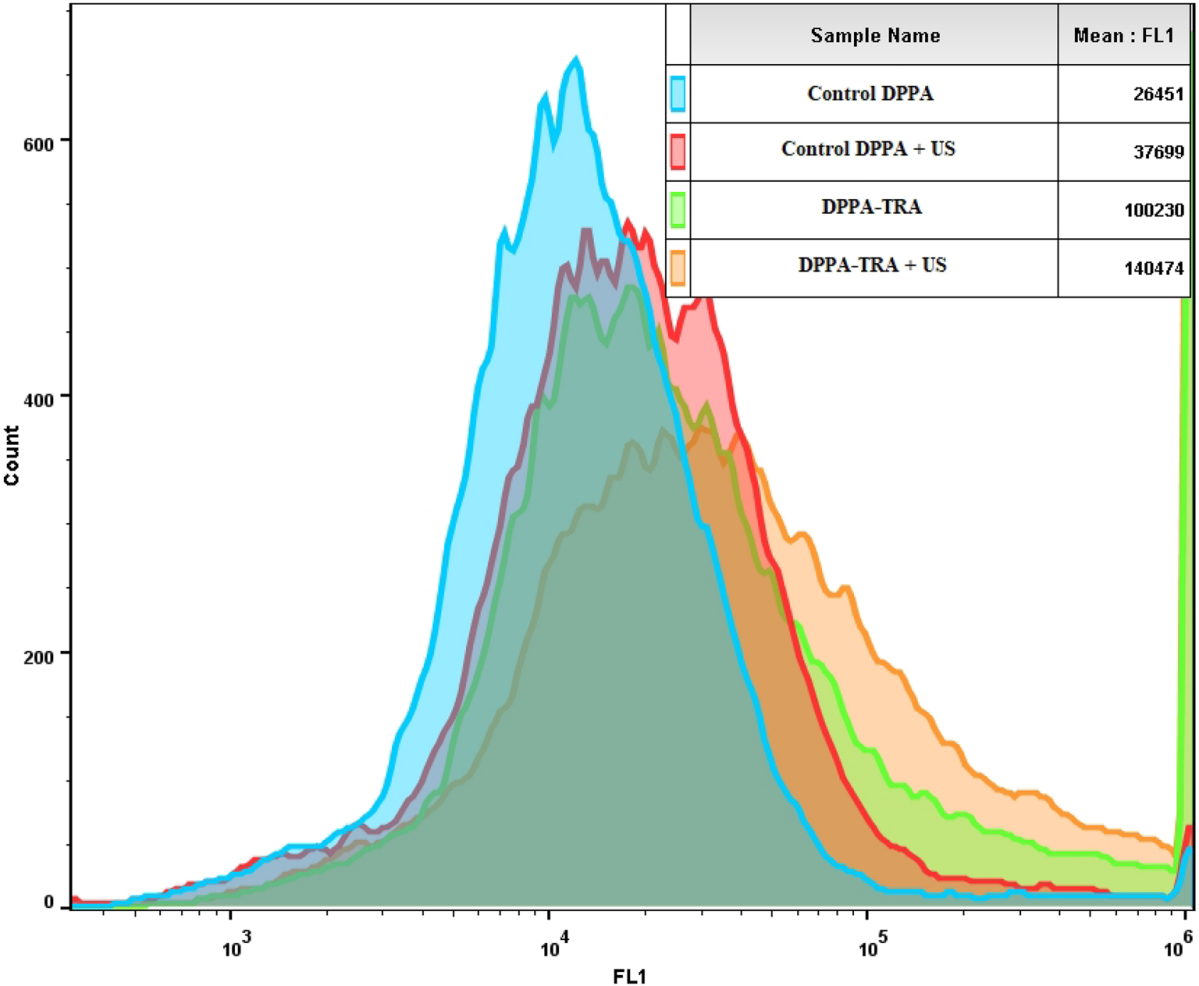
Flow cytometry results showing HCC1954 cellular uptake of calcein after being incubated with control DPPA. control DPPA and DPPA-TRA, before and after drug release triggering using LFUS. An average of 2 × 10^5^ cells/ml cells was analyzed from each sample with calcein fluorescence intensity showed on a four-decade log scale.

Since DPPA-TRA liposomes showed the highest reported calcein uptake by the cells, cells incubated with this formulation were further exposed to LFUS to trigger the release of the encapsulated calcein. As shown in Fig. 7, the exposure to LFUS resulted in enhancing the uptake from both the non-conjugated DPPA and DPPA-TRA, with the highest increase in drug uptake recorded in the cells incubated with DPPA-TRA and sonicated with LFUS showing a 33.4% increase in calcein fluorescence value (140,474 ± 329 A.U.) compared to those incubated with DPPA-TRA with no sonication (100,230 ± 2783 A.U., p = 3.3 × 10^–5^) and a 136.6% increase in calcein uptake compared to those incubated with control DPPA without sonication (26,451 ± 902 A.U., p = 3.04 × 10^–5^).

## Discussion

The use of immunoliposomes functionalized with monoclonal antibodies is a promising approach to improve the specificity of drug-loaded liposomes delivering their load to tumors. Monoclonal antibodies such as Trastuzumab (TRA), conjugated to the surface of liposomes, allow the delivery of toxic chemotherapeutic agents to specifically targeted tumor cells to reduce their adverse side effects significantly. In this case, TRA is only used in small concentrations as a targeting moiety rather than its regular use as an immunotherapy drug to treat hormone-dependent cancers at higher therapeutic doses. TRA was also conjugated to other nanoparticles to enhance their targeting abilities, such as gold nanoparticles^35,36^, dendrimers^37^, and iron oxide nanoparticles^38^. In this study, we used immunoliposomes crafted with PEG and decorated with TRA to target HER2 receptors on the surface of HER2+ cancer cells. We have used four types of phospholipids, including a phospholipid with no head group attached to the phosphate head (DPPA) and three other phospholipids with different head groups attached to their phosphate heads (DPPC, DPPE, and DPPG), to understand the effect of the phospholipid head group on the sensitivity of the pegylated immunoliposomes to LFUS and their uptake by cancer cells.

The phospholipids used here have different transition temperatures; transition temperature (T_m_) is the temperature required to induce a change in the structure of phospholipids from the ordered (gel) phase, where the fatty acid chains are tightly packed, to the disordered (liquid-crystalline) phase, where the fatty acid chains are disordered and loosely packed with higher rates of mobility within the bilayer^39^. Both the acyl chains of the phospholipid, as well as their head groups’ composition, play a role in determining the physical state of the bilayer membrane, including the transition temperature, which is influenced by the length and saturation level of the acyl chain, as well as the structure of the head groups. The phospholipids used in this study have similar fatty acid chains and only differ in their head groups (Fig. 1). Therefore, the difference in their thermal stability is due to the presence of the head group and its chemical composition. All non-conjugated liposomal formulations tested here showed similar sizes (< 200 nm) and polydispersity percentages (< 20%) and only differed in their surface charge, with DPPE and DPPG showing the least and largest negative charge, respectively (DPPE < DPPC < DPPA < DPPG). TRA conjugation was achieved through the post-insertion method, increasing the size and the negative surface charge of all liposomal formulations. Generally, the optimal liposomes’ size ranges between 100 and 200 nm. This allows them to take advantage of the EPR effect and effectively extravasate into tumor tissues while avoiding filtration by both the liver and the spleen^40^. The size of the conjugated formulations prepared in this study was slightly above the recommended size of the nanocarriers. However, depending on the tumor type, the EPR effect operates in the range of 100–400 nm^41^. All the conjugated formulations showed similar stability in FBS; this could be attributed to the added steric stabilization from DSPE-PEG-2000, which is known to enhance the stability of the liposomes by reducing the interactions between the lipid bilayers, as well as, reducing the interactions between the liposomes and serum proteins^42^. Furthermore, all the phospholipids used here contained the same saturated fatty acid chains and the same molar ratio of cholesterol. Generally, combining saturated phospholipids and cholesterol reduces the permeability of the bilayer membrane^43^. This is because saturated fatty acid chains exhibit a linear structure with no bends (each carbon atom binds to the adjacent carbon atoms using two hydrogen atoms), allowing them to be packed tightly^44^. Incorporating cholesterol within the saturated phospholipids strengthens the membrane by forming hydrophobic interactions with the surrounding phospholipid molecules using their head groups as a shield from the water. This will increase stability and lower the membrane's permeability while enhancing its fluidity by preventing the hydrocarbon chains from crystallizing by separating them^45^.

DPPA and DPPE have the highest T_m_ of 65 °C and 63 °C, respectively, while DPPC and DPPG have a similar lower T_m_ of 41 °C. DPPC and DPPE both have nitrogen molecules in their polar head groups (choline and ethanolamine, respectively). Choline molecules are relatively large, with the nitrogen atom bound to three methyl groups. This will lead to reduced hydrogen bonding between the neighboring phospholipids due to the increased distance between the phosphate groups. This results in lowering the thermal stability of DPPC and its T_m_. Charge pairings can also develop among adjacent positively and negatively charged PC head groups. Additionally, water bridges connect the PC head groups within the membrane. Nevertheless, these connections represent dynamic states that undergo multiple cycles of breaking and reforming throughout their lifespan^46^. DPPE, on the other hand, has a smaller head group compared to DPPC. This is because the nitrogen atom is only bound to three hydrogen atoms^47^. This allows the head groups to be attracted to each other through hydrogen bonds adding to the thermal stability and leading to a higher T_m_ of DPPE compared to DPPC phospholipids^48^. Like DPPC, DPPG contains a relatively large functional group (glycerol), which will also increase the distance between the phosphate groups and thus reduce the T_m_. DPPA, on the other hand, only contains phosphoric acid, with no chemical group attached to it, allowing the formation of strong hydrogen bonding between the neighboring phospholipids resulting in a relatively high T_m_^49^. The liposomal formulations used here were generally stable and the encapsulated calcein remained sealed inside the core of these liposomes. However, upon exposure to LFUS, all the tested formulations showed sensitivity to LFUS, triggering the release of their loads. We have previously studied the effect of pegylation on liposomal response to LFUS, as well as high-frequency ultrasound (HFUS) and found that pegylation enhances the sensitivity of DPPC-liposomes to ultrasound compared to non-pegylated liposomes^50^. In this study, we reported an increase in calcein release during the *on* mode of the pulsed LFUS, while no increase in the release was detected during the *off* mode. This indicates that all liposomal formulations maintained their bilayer wall integrity when no LFUS was applied. A previous study by Khvedelidze et al.^49^ has shown that liposomes containing cholesterol can restore their structure, following a transient disruption, as opposed to pure liposomes.

Our results showed that the liposomes prepared using phospholipids with relatively lower T_m_ (DPPC and DPPG) were more sensitive to LFUS compared to those prepared using phospholipids with higher T_m_ (DPPA and DPPE). This is because the energy generated from ultrasound waves causes the hydrophilic load to expand, leading to growing tension between the phospholipids molecules to the point where a critical value is reached. This triggers structural changes and the subsequent release of the encapsulated calcein. In addition to the mechanical effect, ultrasound waves generate a thermal effect. We have observed an increase in temperature to 31 °C following the third pulse of LFUS. Thus, DPPC and DPPG liposomes, which have lower hydrogen bonding between the neighboring phospholipids and lower T_m_ compared to DPPA and DPPE liposomes, showed higher sensitivity to LFUS. In addition, TRA conjugation further enhanced the sensitivity of all the liposomal formulations to LFUS. This could be due to the presence of TRA molecules on the surface of these liposomes, which may destabilize the membrane, further enhancing liposomal sensitivity to the mechanical and thermal effect of the acoustic waves.

We have also investigated the effect of phospholipids’ head group on cellular uptake of pegylated liposomes, which have the same acyl chain length and saturation level (16:0), prior to their conjugation to TRA, and found that calcein, encapsulated inside all the formulations, was taken up by the cells. Generally, liposomes can be engulfed by the cells through the endocytosis process^51^. Overall, the mechanism by which liposomes enter cells depends on factors such as the size, composition, and surface charge of the liposome, as well as the type of the cell and its membrane properties^52^. Furthermore, Some studies have shown that pegylation promotes cellular uptake of liposomes depending on the length of the polymer^53,54^. Our results showed greater calcein uptake by the cells incubated with DPPA liposomes. This could be referred to the fact that phosphatidic acid (PA) enhances lipid uptake by the cells through the enhancement of dynamin-based membrane remodeling, which is involved in receptor-mediated endocytosis. Dynamin is a GTPase that plays a critical role in the formation of clathrin-coated vesicles during endocytosis. PA has been shown to enhance dynamin's activity by binding to its pleckstrin homology (PH) domain and promoting its association with the membrane. This, in turn, leads to the remodeling of the membrane and the formation of endocytic vesicles^55^.

TRA conjugation to all the tested formulations resulted in a significant enhancement of calcein uptake by the cells compared to the non-conjugated formulations. A possible explanation is the increase in the uptake of the TRA-conjugated liposomes by the cells due to the binding of TRA molecules to the targeted HER2 receptors overexpressed on the surface of HCC1954 cells, and their subsequent uptake through the receptor-mediated endocytosis. TRA-DPPA liposomes, which showed the highest uptake of calcein, were then sonicated using LFUS, resulting in further enhancement of calcein uptake by the cells. Studies have shown that ultrasound can enhance the cellular uptake of liposomes by up to 40-fold^56,57^. The mechanism of this enhancement is believed to be related to the ability of ultrasound to promote the release of liposomal contents from within the liposomes by inducing both thermal and mechanical effects on the liposomes. This can cause liposomes to rupture and release their contents. Furthermore, ultrasound can also disrupt cellular membranes by cavitation, which creates transient pores on cellular membranes, allowing liposome entry, a phenomenon known as “sonoporation”^58^.

This study is a proof of concept showing that the efficient internalization of drugs loaded inside pegylated liposomes into tumor cells depends on many factors. This includes the composition of the liposomes, the addition of a suitable targeting moiety combined with the use of LFUS as a triggering mechanism for drug release. Overall, ultrasound is a promising technique for enhancing the cellular uptake of liposomes and improving their effectiveness as drug-delivery vehicles. However, more research is needed to optimize the ultrasound parameters and ensure the safety and efficacy of this technique.

## Conclusion

This work focused on synthesizing TRA-conjugated pegylated liposomes using different phospholipid compositions and studying the effects of LFUS on triggering the release of their payload of calcein. The results showed the TRA-conjugated DPPC liposomes showed the highest sensitivity to LFUS. On the other hand, incubating the cells with TRA-conjugated DPPA liposomes combined with LFUS showed the highest uptake of the loaded calcein by the HER2+ cells. The results showed that combining TRA-conjugated DPPA liposomes with LFUS is a promising formula for achieving the highest drug uptake by cancer cells.

## Data Availability

The datasets generated and/or analysed during the current study are available in the google drive, at https://drive.google.com/drive/folders/1xvYZwOsw-I7jay8zBnnh4ewzbpIN2Jgd.
